# BioTimer assay as complementary method to vortex-sonication-vortex technique for the microbiological diagnosis of implant associated infections

**DOI:** 10.1038/s41598-019-44045-1

**Published:** 2019-05-17

**Authors:** Luigi Rosa, Maria Stefania Lepanto, Antimo Cutone, Francesca Berlutti, Massimiliano De Angelis, Vincenzo Vullo, Claudio Maria Mastroianni, Piera Valenti, Alessandra Oliva

**Affiliations:** Department of Public Health and Infectious Diseases, University of Rome La Sapienza, Rome, Italy

**Keywords:** Applied microbiology, Bacterial infection, Biofilms

## Abstract

To enumerate bacteria adherent to medical devices, Vortex-Sonication-Vortex Method (VSVM) and BioTimer Assay (BTA) have been applied. VSVM counts detached microorganisms whereas BTA enumerates adherent microorganisms through microbial metabolism. However, the limitation of VSVM consists in incomplete detachment of adherent microorganisms while BTA is unable to identify microbial genera and species. Herein, the combined use of VSVM and BTA for the diagnosis and enumeration of adherent microorganisms causing implant-associated-infections (IAIs) is reported. Over 2016–2018, 46 patients with IAIs were enrolled and their 82 explanted devices were submitted firstly to VSVM and then to BTA. VSVM plus BTA detected microorganisms in 39/46 patients (84.7%) compared with 32/46 (69.5%) and 31/46 (67.3%) by VSVM and BTA alone, respectively. Likely, combined methods led to microorganism detection in 54/82 devices (65.9%) compared with each method alone [43/82 (52.4%), 44/82 (53.6%) for VSVM and BTA, respectively]. The combination of both methods (concordance 75.6%) raised the sensitivity of microbial analysis in IAIs compared with either VSVM or BTA alone, thus representing a simple and accurate way for the identification and enumeration of microorganisms adherent on devices. Moreover, BTA reagent applied in a new apparatus allowed also the enumeration of the microorganisms adherent on different segments of cardiac electrodes, thus contributing to define IAIs pathogenesis.

## Introduction

Implant-associated infections (IAIs) have been increasing worldwide and are associated with high morbidity and mortality.

The IAIs are a real challenge for physicians due to the wide variety of presenting symptoms and their chronic and relapsing nature as well as to the complex diagnosis of microbial biofilm colonizing the implants^1–3^.

To obtain a microbiological diagnosis, complete implant removal is required and the analysis is based on the detachment of microorganisms followed by the count of Colony Forming Units (CFUs). The CFUs method is the sole validated method for enumerating planktonic microorganisms, while it is unreliable for sessile microbial biofilm on abiotic surfaces^4,5^. Of note, this method gives up to 30% of false-negative results because microorganisms are encased in biofilm thus hindering microbial detachment and detection^6,7^. In addition, the diagnostic sensitivity is influenced by the empiric antimicrobial therapy, administered before the implant removal, which leads to a further lowering microbial detection rate.

To overcome this drawback, microbiologists have set up several semi-quantitative methods. One of these is actually based on the roll-plate technique^8,9^, which counts the microorganisms detached from the device surface, but it does not determine the intraluminal colonization in case of catheters’ analysis. Other methods are based on the detachment of microbial biofilm from implants by Vortex^10^ or by Vortex-Sonication-Vortex (VSV) treatment^11–14^. However, although the VSV method (VSVM) is widely applied, it is not universally accepted as gold standard to enumerate microorganisms in biofilm^5^. As a matter of fact, VSVM shows some limitations as (i) not all adherent microorganisms in biofilm might be detached; (ii) the sonication treatment could affect microbial viability leading to erroneous counts; (iii) the CFU counts are referred to the number of planktonic detached microorganisms rather than the total number of adherent microorganisms. Another method to detect microbial biofilm is related to the impedance measurement^15^ which, nevertheless, does not distinguish neither the microbial genera or viability. Among the molecular methods, the Real time PCR has been applied mainly to *Staphylococcus aureus* biofilm^16^ and not all microorganisms in biofilm might be lysed.

For all these limits, the actual and reliable microbial count has been carried out through the detection of microbial metabolic products. These methods, defined as indirect assays, need specific correlation lines in which metabolic product is related to the number of microorganisms in adherent, aggregate or biofilm lifestyle^4,17–19^. Among these, the detection of ATP synthesis has been related to the number of viable microorganisms^17^, but the limit of this method consists in the uncertainty that all microorganisms in biofilm are properly lysed. Another indirect method is named BioTimer Assay (BTA). It determines the actual number of microorganisms in free, aggregated, adherent and biofilm lifestyle using an original reactive containing Phenol Red or Resazurin as indicators. The Phenol Red changes colour from red to yellow indicating the presence of fermenting microorganisms, while Resazurin switches from violet to pink detecting non-fermenting ones^20^. The time required for indicators’ switching is correlated to the initial number of microorganisms (N0) through a genus-specific correlation line described by the following equation: t = log (1 + *a*/N0)/*k* where *k* is growth rate and *a* is a function of the metabolic product responsible for the indicator switching^4^. As there is no validated method to count microorganisms in aggregated, adherent and biofilm lifestyle, the correlation lines were designed using planktonic microorganisms enumerated by CFUs method. Accordingly, the number of microorganisms detected by BTA is referred as planktonic equivalent^21^.

BTA is low-cost, easy to perform and doesn’t require samples’ manipulation. In the last years, it has been widely applied to (i) quantitatively evaluate bacteria adherent to polyelectrolyte HEMA-based hydrogels^4^, (ii) enumerate microorganisms on foods^22^, (iii) count *Staphylococcus* spp. in biofilm^21^, (iv) enumerate bacteria in medical devices^23–25^ and (v) detect the number of living microorganisms in biofilm on central venous catheters^20^.

Here, we report the use of BTA for the diagnosis of IAIs, including cardiovascular implantable electronic devices infections (CIED-I), peripherally inserted central catheter-associated infections (PICC-I), port-a-cath infections (PORT-I), central venous catheters infections (CVC-I) and ureteral stent infections (US-I). A new BTA reagent containing both Phenol Red and Resazurin Indicators (PhRRIs) was set-up in order to enumerate microorganisms. In particular, this new reagent is able to selectively distinguish fermenting from non-fermenting microorganisms through the indicators’ colour switch from violet-to-yellow and from violet-to-orange, respectively.

Overall, even if we have developed a more suitable reagent, BTA is unable to determine microbial genus or species. On the other hand, also VSVM, allowing to identify microbial genera and species, possesses, anyhow, limits already reported such as not complete biofilm detachment, under-estimated microbial number and false-negative results.

To overcome the intrinsic limits of both methods, here we suggest to contemporary apply the innovative BTA and the standard VSVM to analyse samples from subjects with CIED-I, PICC-I, PORT-I, CVC-I and US-I.

Moreover, in order to have additional insights on the pathogenesis of CIED-I, a new analytical apparatus, able to detect the different degrees of microbial colonization along a ‘non-cuttable’ medical device (such as cardiac leads), was firstly applied to analyse the atrial and the ventricular lead collected from a 66-years old woman with CIED-I.

## Results

### Study population

Over the study period, a total of 46 patients with implant infections were enrolled (23 subjects with CIED-I, 21 with PICCs/PORT/CVC-I, 2 with US-I) (Table 1). In particular, among 23 patients with CIED-I, 5 presented with lead-associated endocarditis and 18 with pocket infections.

**Table 1:** Study population.

	Total patients No. 46
**Type of implants**	
CIED	23/46
PICC/PORT/CVC	21/46
US	2/46
**Positive blood cultures**	26/46 (56.5%)
Lead-associated endocarditis	5/5
Pocket infections	0/18
PICC-I/PORT-I/CVC-I	21/21
US-I	0/2
**Negative blood cultures**	20/46 (43.5%)
Lead-associated endocarditis	0/5
Pocket infections	18/18
PICC-I/PORT-I/CVC-I	0/21
US-I	2/2
**Positive microbiological diagnosis by VSVM**	32/46 (69.5%)
Lead-associated endocarditis	1/5
Pocket infections	14/18
PICC-I/PORT-I/CVC-I	17/21
US-I	0/2
**Negative microbiological diagnosis by VSVM**	14/46 (30.5%)
Lead-associated endocarditis	4/5
Pocket infections	4/18
PICC-I/PORT-I/CVC-I	4/21
US-I	2/2
**Positive microbiological diagnosis by BTA**	31/46 (67.3%)
Lead-associated endocarditis	4/5
Pocket infections	14/18
PICC-I/PORT-I/CVC-I	12/21
US-I	1/2
**Negative microbiological diagnosis by BTA**	15/46 (32.7%)
Lead-associated endocarditis	1/5
Pocket infections	4/18
PICC-I/PORT-I/CVC-I	9/21
US-I	1/2
**Overall positive microbiological diagnosis (VSVM plus BTA)**	39/46 (84.7%)

Cardiovascular implantable electronic devices infection (CIED-I); peripherally inserted central catheter infection (PICC-I); port-a-cath infection (PORT-I); central venous catheter infection (CVC-I); ureteral stent infection (US-I); Vortex-Sonication-Vortex method (VSVM); BioTimerAssay (BTA).

Blood cultures were performed in all subjects. The hemocultures were positive (bacteraemia) in 26/46 (56.5%) patients of which 5 with lead-associated endocarditis and 21 patients with PICC-I/PORT-I/CVC-I. The hemocultures were negative in 20/46 (43.5%) patients of which18 with pocket infections, and 2 with US-I (Table 1).

Overall, microbial detection with both VSVM and BTA was positive in 39 out of 46 patients (84.7%) compared with 32/46 (69.5%) and 31/46 (67.3%) by VSVM and BTA alone, respectively (Table 1), distributed as follows: 24 VSVM+/BTA+, 7 VSVM−/BTA−, 8 VSVM+/BTA−, 7 VSVM−/BTA+, respectively. In detail, as for CIED-I (n = 23), VSVM and BTA were both positive and negative in 13 and 3 subjects, respectively, whereas 2 were VSVM+/BTA− and 5 VSVM−/BTA+. When considering subjects with PICC-I/PORT-I/CVC-I (n = 21), 11 were VSVM+/BTA+ whereas 3 were VSVM−/BTA−, 6 VSVM+/BTA− and 1 VSVM−/BTA+, respectively. As for subjects with US-I (n = 2), 1 was VSVM−/BTA− and 1 VSVM−/BTA+. The devices’ positive microbiological diagnosis with VSVM was obtained in 32/46 (69.5%) patients of which 15/23 with CIED-I (1 with lead-associated endocarditis, 14 with pocket infections), 17/21 with PICC-I/PORT-I/CVC-I and 0/2 with US-I (Table 1). Sterile devices were found in the remaining 14 patients (30.5%).

The devices’ BTA analysis highlighted a positive microbiological diagnosis in 31/46 (67.3%) patients of which 18/23 with CIED-I (4/5 with lead-associated endocarditis, 14/18 with pocket infections), 12/21 with PICC-I/PORT-I/CVC-I and 1/2 with US-I (Table 1). Sterile devices were found in the remaining 15 patients (32.6%).

Summarizing, among subjects with lead-associated endocarditis (n = 5), VSVM led to a definite diagnosis of infection in only 1 case, while BTA in 4/5 subjects including the one detected by VSVM. The pocket infections were confirmed in 14 out of 18 subjects by both methods. The infections of 21 subjects with PICCs/PORT/CVC were confirmed in 17/21 by VSVM while in 12/21 by BTA. The infections of 2 patients with USs were confirmed in 0 patients by VSVM while in 1 patient by BTA.

In case of microorganism’s growth from both VSVM and BTA, all the microorganisms matched at class level.

### Removed devices

The 46 enrolled patients carried a total of 82 devices: 16 generators, 42 atrial/ventricular leads, 19 PICCs, 1 port-a-cath, 1 CVC and 3 USs (Table 2). The microbiological analysis was contemporary performed with both VSVM and BTA and it was highlighted the infection in 54 out of 82 (65.9%) collected devices. However, considering VSVM and BTA alone, 43 and 44 devices resulted infected, respectively. Therefore, the contemporary analysis carried out with both methods allows to detect more precisely the infected devices (Table 2). In Table 2, the infection of each device detected by both methods (VSVM and BTA) is reported.

**Table 2:** Devices collected and analyzed by Vortex-Sonication-Vortex method (VSVM) and BioTimer Assay (BTA).

Devices collected	Total No. of devices	Positive microbiological diagnosis VSVM plus BTA	Total concordance
	82	54/82 (65.9%)	62/82 (75.6%)
Generators	16/82	12/16	13/16
Leads	42/82	21/42	34/42
PICCs	19/82	16/19	14/19
Port-a-cath	1/82	1/1	0/1
CVC	1/82	1/1	0/1
USs*	3/82	2/3	1/3

Leads: atrial or ventricular lead; peripherally inserted central catheters: PICCs; central venous catheter: CVC; ureteral stents: USs.

*One patient had two subsequent US-Is.

In particular, among a total of 16 generators, 12 resulted infected of which 9 positive by both methods, 1 positive only by VSVM and 2 only by BTA. Among a total of 42 leads, 21 resulted infected of which 13 positive by both methods, 3 positive only by VSVM and 5 only by BTA. Among a total of 19 PICCs, 16 resulted infected of which 11 positive by both methods, 5 positive only by VSVM and 0 only by BTA. Regarding port-a-cath and CVC, VSVM and BTA detected microorganisms on CVC and port-a-cath, respectively. Among 3 USs from 2 patients, 1 was sterile for both method and 2 yielded microbial cells only by BTA (Table 2).

The total concordance between VSVM and BTA corresponded to 62/82 (75.6%) (Table 2).

### Enumeration of microorganisms

The number of microorganisms was obtained with both VSVM and BTA. The differences between these two methods consist in the enumeration of the sole detached microorganisms by VSVM and in the number of the adherent plus detached microorganisms by BTA. Moreover, the total analytical time required by BTA was shorter than the one by VSVM: 1407 versus 2928 hs.

The microbial enumeration by BTA can be influenced by the previous VSV treatment, which completely or partially detaches microorganisms, thus leading to a different number of remaining adherent microorganisms to be detected by BTA. For this reason, we set up a new procedure in which VSVM is carried out in a new BTA reagent instead of saline solution^12–14^. In this way the device is immerged in the PhRRIs reagent, which, after VSVM, is used for classical microbiological and BTA analyses where the immerged device is incubated at 37 °C without any further manipulation. It is important, however, to underline that in PICCs analysis the reagent is completely taken for classical microbiological analysis, thus interfering with the results deriving from BTA (see Materials and Methods).

**Figure 1:**
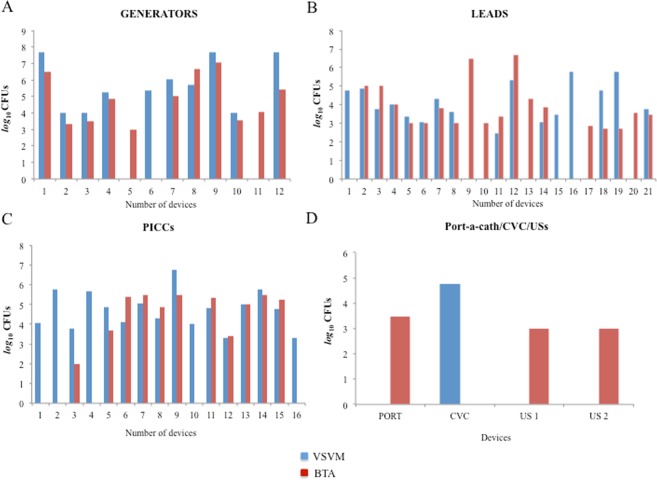
Enumeration of microorganisms by Vortex-Sonication-Vortex method (blue) and BioTimer Assay (red) colonizing generators (Panel A), leads (Panel B), peripherally inserted central catheters (PICCs) (Panel C), port-a-cath (PORT), central venous catheters (CVCs) and ureteral stents (USs) (Panel D). The sterile devices were omitted from the graphs.

The number of microorganisms detected by each method is reported in Fig. 1 in which the devices sterile with both methods, such as 4 generators, 21 leads, 3 PICCs and 1 US, are omitted.

As shown in Fig. 1A, VSVM determined a higher number of microorganisms compared to BTA in 3 generators (generators 1, 7, 12) and an almost similar number detected by both methods in 6 generators (generators 2, 4, 5, 8, 9, 10). The discordances between two methods were evidenced in generator 6, resulting infected only by VSVM, and in generators 4 and 11 considered infected only by BTA.

In Fig. 1B, the enumeration of microorganisms colonizing leads is reported. VSVM assayed a higher number of microorganisms only in 2 samples (leads 18, 19), while BTA in 3 samples (leads 3, 11, 14). In 8 leads the enumeration was almost similar with both methods (leads 2, 4, 5, 6, 7, 8, 12, 21). Among 8 discordances 3 samples were considered infected only by VSVM (leads 1, 15, 16), while 5 resulted infected only by BTA (leads 9, 10, 13, 17, 20).

The Fig. 1C summarizes the PICCs analysis. The microbial enumeration was almost similar with both methods in 5 PICCs (PICCs 7, 8, 12, 13, 14) while a higher number of infecting microorganisms was detected in PICCs 3, 5, 9 by VSVM and in PICCs 6, 11, 15 by BTA. The discordances were 5 because only VSVM identified the infections in PICCs 1, 2, 4, 10, 16 and 0 by BTA. Discordant results were detected in 5 samples (PICCs 1, 2, 4, 10, 16) resulting infected only by VSVM, indicating that VSVM is very efficient in the complete bacteria detachement adherent on PICCs In Fig. 1D, the analyses of 1 port-a-cath, 1 CVC and 2 USs are reported. As shown, BTA detected the infections in 3 out 4 samples (1 port-a-cath and 2 USs) while VSVM only in 1 CVC.

### Identification of microorganisms

The fermenting microorganisms detected by BTA corresponded to *Staphylococcus aureus*, *Staphylococcus epidermidis*, *Candida albicans*, *Serratia marcescents*, *Corynebacterium striatum*, *Klebsiella pneumoniae* and *Nocardia cyriacigeorgica*, as identified by Vitek-2. Non-fermenting bacteria by BTA corresponded to *Pseudomonas aeruginosa* identified by Vitek-2.

### Insight on CIED-I pathogenesis through the use of new analytical apparatus produced by a 3D printer

A 66-years old woman, carrier of generator and ventricular and atrial leads, was hospitalized for inflammation at pocket level and cutaneous fistula. Blood cultures (three specimens performed every 30 min) were negative for microbial growth and echocardiography evidenced absence of vegetation.

The positive microbiological diagnosis by VSVM showed total detached microorganisms from the generator corresponded to 5 × 10^5^ CFUs and from ventricular and atrial leads to 2 × 10^5^ and 3 × 10^2^ CFUs, respectively (Table 3). The classical identification method, carried out on planktonic microorganisms detached by VSVM, indicated after 48 hs the presence of *P. aeruginosa* in agree with non-fermenting strain detected by BTA. In particular, in the microbiological diagnosis by BTA, PhRRIs switched from violet-to-orange after 330 min indicating a total of 5 × 10^6^ non-fermenting microorganisms adherent to the generator. This value was higher than the one detected by VSVM (Table 3) even if the generator was previously treated in saline solution by VSVM.

**Table 3:** The new analytical apparatus with BTA reagent was used for the detection of the adherent bacteria along different atrial and ventricular lead segments in a 66-years old patient carrying generator, atrial and ventricular leads. The comparative analyses were carried out by VSVM.

	Total CFUs by VSVM	Total equivalent CFUs by BTA
Generator	5.0 × 10^5^	5.0 × 10^6^
**Atrial lead**	3.0 × 10^2^	2.4 × 10^4^
Proximal segment	/	2.0 × 10^4^
Middle segment	/	4.0 × 10^3^
Distal segment	/	4.0 × 10^3^
**Ventricular lead**	2.0 × 10^5^	5.0 × 10^6^
Proximal segment	/	2.0 × 10^6^
Middle segment	/	1.5 × 10^6^
Distal segment	/	1.5 × 10^6^

Colony Forming Units: CFUs.

**Figure 2:**
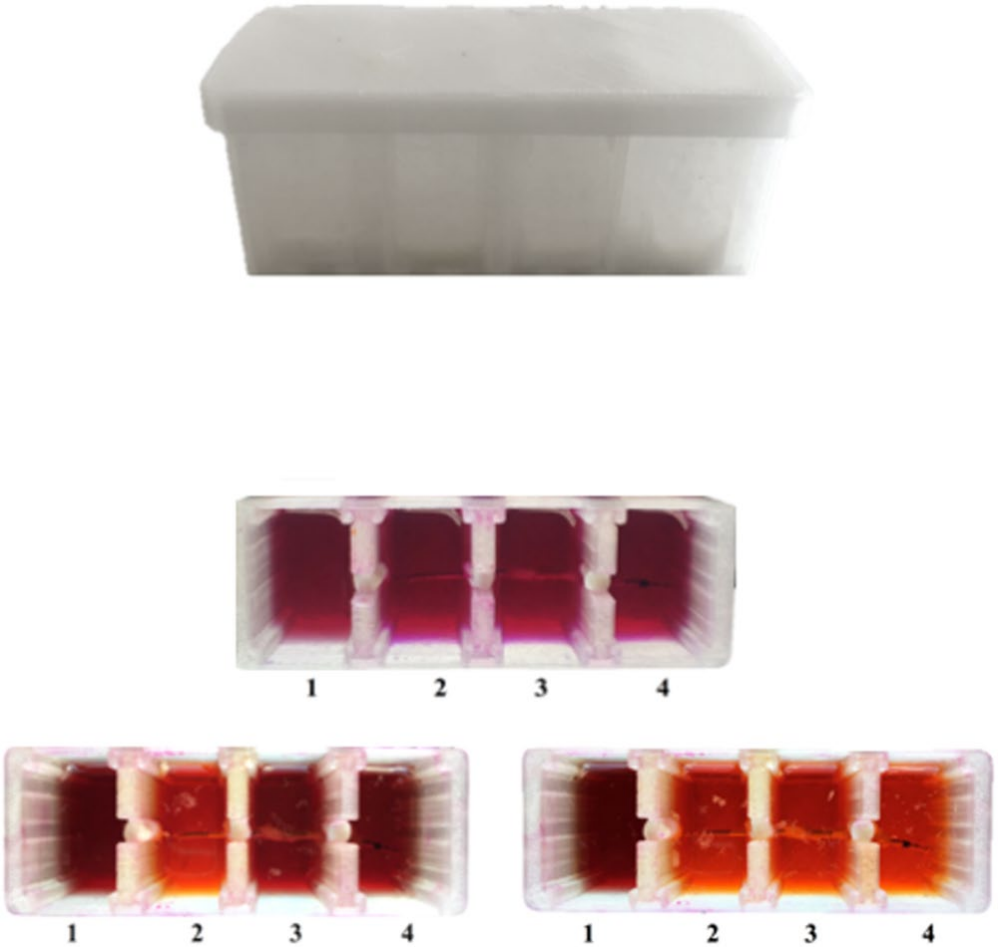
New analytical apparatus for BioTimer Assay. The compartment 1 was used as sterility control; compartments 2–4 were used for the analysis of atrial or ventricular leads horizontally lodged. Each compartment was isolated with paraffin and filled with Phenol Red and Resazurin Indicators (PhRRIs). In the compartment 2 the proximal segment, in the compartment 3 the middle segment and in the compartment 4 the distal segment of lead were analyzed, respectively. The PhRRIs colour switch, from violet-to-orange, indicated the presence of non-fermenting adherent microorganisms. The number was calculated by using the correlation line showed in Fig. 4.

Regarding the analysis by BTA of both leads, it was performed through a new analytical apparatus (Fig. 2). PhRRIs switched from violet-to-orange after 640 min for the proximal segment of the atrial lead, indicating the presence of 2.0 × 10^4^ CFU non-fermenting adherent microorganisms, whereas middle and distal atrial segments required both 765 min for colour switch, showing a total of 2.0 × 10^3^ CFU adherent microorganisms (Table 3). The proximal segment of ventricular electrode required 380 min for colour switch corresponding to a total of 2.0 × 10^6^ adherent microorganisms, while the middle and distal segments both required 400 min for colour switch, corresponding to a total of 1.5 × 10^6^ adherent microorganisms (Table 3).

## Discussion

**Figure 3:**
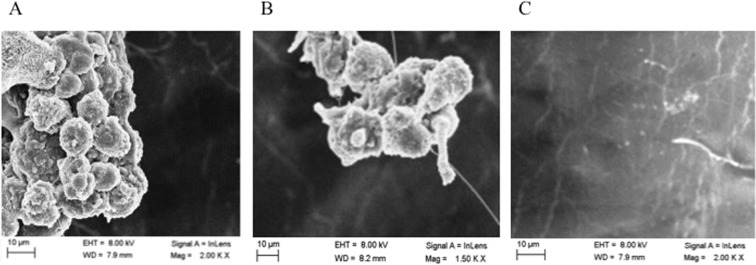
Scanning electron microscopy images showing the different amounts of detached biofilm (Panel A and B) or microorganisms (Panel C) present in the solution after Vortex-Sonication-Vortex method obtained from a representative device.

VSVM, to count microbial cells on the devices through CFUs counts and to allow their identification, must detach the microorganisms. Although comparison of different methods has been widely reviewed in the literature, there is currently no uniform gold standard for detection and quantification of bacteria in biofilm^5^. For instance, VSVM detaches with different efficacy and releases in solution after the treatments different amounts of biofilm and/or microorganisms (Fig. 3), depending from the layer and the age.

Conversely, BTA does not require any manipulation of the sample because it is based on the indirect count of microorganisms through their metabolic products. On the other hand, BTA possesses the limit in the identification of microbial genera and species.

In order to overcome the intrinsic limits of VSVM and BTA, here we propose to carry out the analysis of explanted devices firstly with VSVM and then with BTA for the diagnosis of IAIs and to compare the results obtained with both. This combination involves following changes: (i) the substitution of the saline solution utilized for VSVM with the BTA reagent; (ii) the creation of a new BTA reagent, PhRRIs, able to contemporary distinguish fermenting from non-fermenting microorganisms; (iii) the enumeration by BTA of both detached and still adherent microorganisms after VSVM. These modifications guarantee the actual enumeration of microorganisms thus avoiding the false-negative results supplied by VSVM.

Since BTA is still not validated, VSVM must be the first analytical step. However, it might influence the subsequent analytical data obtained by BTA. The sole substitution of saline solution with BTA reagent only partially avoids the false-negative results by BTA. In fact, when the classical microbiological analysis requires the removal of all the volume of solution obtained by VSVM, BTA can enumerate only the remained adherent microorganisms. Thus, if VSVM detaches all microorganisms and the whole pellet is used for classical microbiological analysis, BTA furnishes false-negative results, as it happened in our study with PICCs.

This study has been performed on 46 patients with IAIs with a total of 82 devices. Overall, we observed that not all positive blood cultures corresponded to all colonized devices from each patient (Tables 1, 2). This is an expected result, given that pocket infections as well as US-I are not usually associated with concomitant bloodstream infection; on the other hand, in case of PICC-I/PORT-I/CVC-I, blood cultures are positive in the majority of patients.

Regarding the total concordance between VSVM and BTA, it ranges from 74 to 82% depending from the type of devices (generators, leads and PICCs) (Table 2) as well as from the experimental procedure described above. The number of PORT, CVC and USs is so low to be not statistically relevant to detect the concordance (Table 2).

We demonstrated that the combination of both methods was able to raise the sensitivity of microbial analysis in IAIs in comparison with VSVM and BTA alone. In fact, in absence of a gold standard method for detection and quantification of bacteria in biofilm, the combination of VSVM and BTA can provide a simple, accurate and reproducible way for the enumeration and identification of microorganisms adherent on devices. Furthermore, we should take into account that one of the advantages of BTA over VSVM is represented by the shorter analytical time required. Thus, according to the results of our study, the potential advantage of using the VSVM/BTA combination resides on the possibility that in additional 7 patients (15%) a microbiological diagnosis can be obtained, in comparison with each technique alone. This finding is of crucial importance, since a correct microbiological diagnosis of IAIs might have consequences in terms of reducing the use of broad-narrow antimicrobials and using targeted therapies, in line with antimicrobial stewardship principles.

Among all infections related to implants, important complications in terms of high morbidity and mortality have been observed in cardiac device implantation. In this respect, the quantification of adherent microorganisms along the lead segments provides important information for pathogenesis understanding. For this purpose, we also designed a new apparatus able to detect the different degrees of microbial colonization along leads segments by BTA (Fig. 2).

PhRRIs and the new analytical apparatus allow us to enumerate total adherent microorganisms to each segment of the lead, making consistent the comprehension of the CIED-I pathogenesis. In particular, here, the higher total number of microorganisms adherent to the proximal lead segment demonstrated that the infection started from the generator, as afterwards confirmed by the absence of lead-associated endocarditis. Thus, the pathogenesis of such infection resided on local perioperative wound contamination rather than on hematogenous source.

Even if this new apparatus requires to be applied on a larger number of devices, it is the first promising method that determines the pathogenesis of cardiac device infection and might be applied in devices other than CIED, such as PICCs, where the actual number of adherent microorganisms to the abiotic surface is not regularly distributed.

The main limitation of the study is the lack of samples collected for reasons other than infections; thus, appropriate analyses regarding specificity, negative and positive predictive values of VSVM and BTA could not be performed. Another limitation is the low number of USs included in the study; however, although only few devices were collected, we could demonstrate the feasibility of both techniques on all the collected materials, including the extra-vascular ones.

In summary, our study demonstrates that VSVM plus BTA could be a reliable tool for the microbiological diagnosis in IAIs. In particular, the combination of both methods allows i) to define infected a device in a less time; ii) to enumerate an actual number of detached and adherent microorganisms on implants; iii) to eliminate false-negative results, and iv) to diagnose the pathogenesis of infections using a new analytical apparatus.

## Materials and Methods

### Study population

The study was conducted in a large Italian academic hospital over a 2-years period (2016–2018) and prospectively enrolled a total of 46 patients with IAIs who were hospitalized at Policlinico Umberto I, Sapienza University of Rome.

Device-related endocarditis was diagnosed according to the modified Duke criteria^26,27^, whereas clinical diagnosis of pocket infection was based on the local signs of inflammation as erythema, warmth, fluctuance, wound dehiscence, tenderness, and purulent drainage. Diagnosis of PICC-I, PORT-I, CVC-I and US-I was made according to the international guidelines^28^.

The infected implants (CIEDs, PICCs, PORT, CVC and USs) were removed as part of infection treatment. Implants’ removal was performed under aseptic condition bedside for PICCs, PORT and CVCs, in the Cardiac Electrophysiology Laboratory for CIEDs and in Urology Department for USs. In addition, blood cultures were performed in all the patients.

Inclusion criteria were all consecutive patients with IAIs requiring the device removal as part of infection management during the study period of time and the enrollment was by convenience sampling. Exclusion criteria were absence of asepsis during device collection.

Demographic, clinical, microbiological, and laboratory data were recorded for each patient. The study was approved by the institutional review board (Section of Infectious Diseases, Department of Public Health and Infectious Diseases, Sapienza University of Rome, n. 2968, November 14th 2013). All study participants gave informed written consent. All experiments were performed in accordance with guidelines and regulations.

### Analysis of removed devices

#### Vortex-sonication-vortex

All the collected implants (generators and leads for CIEDs; PICCs; PORT; CVC and USs) were submitted to cultural analysis after VSV modified treatment. In particular, in order to permit the simultaneous analysis by VSVM and BTA, the devices were immerged in the new BTA reagent (PhRRIs, see BTA section) instead of the standard saline solution.

Briefly, each collected device was completely covered with different volumes of sterile PhRRIs reagent (50 ml for generators, 6 ml for leads, 15 ml for PICCs, PORT, CVC and USs).

Successively, each device was vortexed for 30 sec, sonicated for 5 min (frequency 40 ± 2 kHz and power density 0.22 ± 0.04 W/cm^2^, BANDELIN electronic GmbH & Co. KG) and vortexed again for 30 sec. After VSV treatment, different amount of suspensions (10 ml for generators, 2 ml for leads and 5 ml for PORT, CVC and US) were taken and centrifuged at 3200 rpm for 15 min. For PICC, total volume of the suspension (15 ml) was removed and centrifuged at 3200 rpm for 15 min.

The pellet obtained was re-suspended in 1 ml of saline solution, and to count microbial cells, serial 10-fold dilutions were plated onto Mueller Hinton agar plates and the colonies forming units after 24 hs of incubation were counted and expressed as CFU/ml.

### BioTimer Assay

**Figure 4:**
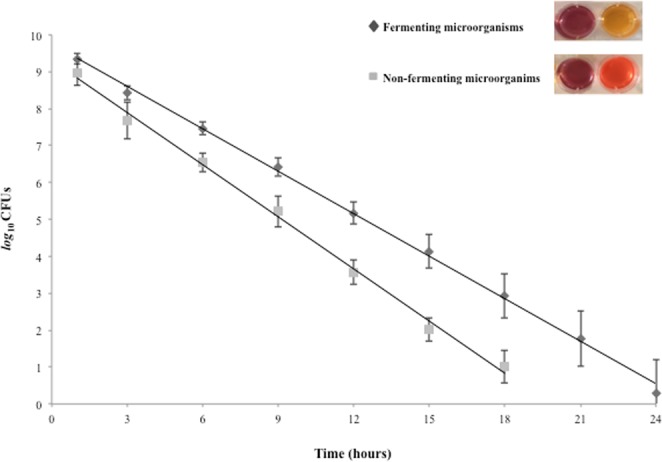
Correlation lines obtained by BioTimer Assay with the reagent containing both Phenol Red and Resazurin Indicators (BTA-PhRRIs) using fermenting (colour switch from violet-to-yellow) and non-fermenting (colour switch from violet-to-orange) microorganisms. The correlation lines show the relationship between the time (X axis) required for colour switch and the initial number of planktonic equivalent microorganisms per milliliter (Y axis).

The PhRRIs reagent was prepared as follows: 3.7 g of Brain Heart Infusion (Oxoid LTD, UK) were dissolved in 940 ml of distilled water. After sterilization at 121 °C for 15 min, 50 ml of 10% filtered glucose solution, 10 ml of filtered 0.25% Phenol Red (Sigma Aldrich, Italy) and 10 ml of 0.1% filtered Resazurin solution (Sigma-Aldrich, Italy) were added and the pH was adjusted to 7.2 ± 0.1. The final reagent appeared clear and violet. The colour change was monitored every 30 min until 24 hs. The time required for colour change of PhRRIs reagent was correlated to initial microbial concentration by correlation lines. Briefly, serial two-fold dilutions of distinct planktonic overnight broth cultures of fermenting and non-fermenting microorganisms were performed in 24-well plates (BD, Italy) at 37 °C in a total volume of 1 ml of PhRRIs reagent and simultaneously counted using the CFUs method. The colour change of the PhRRIs reagent, due to metabolism of fermenting microorganisms was measured through the change of OD values at 420 nm (yellow) and at 558 nm (orange) for non-fermenting microorganisms at regular time intervals (30 min). The time required for PhRRIs colour switch was plotted versus the mean values of the log_10_ of CFUs/ml for fermenting and non-fermenting microorganisms (Fig. 4). The equations and the linear correlation coefficients describing the correlation lines were: y = −0.3838x +9.7543 R^2^ = 0.9982 for fermenting and y = −0.4709x +9.3043 R^2^ = 0.9965 for non-fermenting microorganisms.

After the PhRRIs withdrawal to be used for VSVM, the generators, leads, PORT, CVC and USs immerged in the remaining PhRRIs reagent were incubated at 37 °C without any further manipulation of the sample. This enumeration takes into account both the total number of detached microorganisms and the total remaining adherent microorganisms after VSVM. Conversely, all PICCs were newly immerged in the PhRRIs supernatant, without pellet, obtained from the centrifugation and incubated at 37 °C with no further manipulation.

### Microbial identification

The microorganisms were identified using conventional methods (VITEK-2, Bio-Merieux, Marcy l’Etoile, France). In particular, the pellet obtained was re-suspended, plated on different selective media, aerobically and anaerobically incubated at 37 °C and checked daily up to 10 days.

### Analytical apparatus produced by a 3D printer

The new analytical apparatus (Fig. 2), used on atrial and ventricular leads removed from a 66-years old woman with CIED-I, was designed and produced by a 3D printer (Galileo Smart, Kentstrapper srl, Florence, Italy) in order to evaluate the degree of microbial colonization along the different segments of the device and, therefore, to contribute to the comprehension of IAIs pathogenesis. The polyethylene terephthalate (Kentstrapper srl, Florence, Italy) used to build the apparatus is heat-resistant, transparent and easy to sterilize with 5% of sodium hypochlorite. As reported in Fig. 2, the apparatus was designed as a unique chamber with 4 compartments.

After VSVM, each lead was horizontally lodged in the apparatus in sterile condition. Each compartment was isolated with paraffin and filled with 20 ml of sterile PhRRIs. The compartment 1 was specific to control the sterility of PhRRIs, while the other compartments (2, 3 and 4) were dedicated to the enumeration of microorganisms remaining adherent after VSVM along the segments of the lead (Fig. 2). The apparatus was incubated at 37 °C and monitored each 30 min for colour switch.

### Scanning electron microscopy

To prove the presence of biofilm as well as microorganisms detached by VSVM, the scanning electron microphotographs were performed on a VSVM suspension deriving from a representative device and acquired by a scanning electron microscope (SEM XL30, FEI Company, The Netherlands) after fixation of the samples with 2.5% glutaraldheyde at 4 °C for 24 hs. The sample was treated with 1% OsO_4_ for 2 hs and then dehydrated with increasing percentage of ethanol from 40 to 95% and coated with colloidal gold.

### Statistical analysis

Values of microbial cell counts were given as means ± standard errors of the means (SEMs). Categorical variables were compared by using the McNemar test and *P* value of < 0.05 was considered statistically significant. Statistical analyses were performed using STATA (version 9) software (STATA Corp. LP, College Station, TX).

For the correlation lines mean values and standard deviations were calculated on whole data sets obtained from at least five independent experiments. Correlation lines, obtained by linear regression analysis and linear correlation coefficients, were calculated from the equation:

$${\rm{r}}=({\rm{n}}{\rm{\Sigma }}\mathrm{xy}-{\rm{\Sigma }}{\rm{x}}{\rm{\Sigma }}{\rm{y}})/({\rm{sqrt}}(({\rm{n}}{\rm{\Sigma }}{\rm{x}}2-({\rm{\Sigma }}{\rm{x}}){\rm{2}})({\rm{n}}{\rm{\Sigma }}\mathrm{y2}-({\rm{\Sigma }}{\rm{y}}){\rm{2}}))).$$

## Data Availability

The data used to support the findings of this study are available from the corresponding author upon request.
